# Left atrial pathological degeneration assessed by integrated backscatter transesophageal echocardiography as a predictor of progression to persistent atrial fibrillation: Results from a prospective study of three-years follow-up

**DOI:** 10.1186/1476-7120-10-28

**Published:** 2012-06-29

**Authors:** Tomoki Kubota, Masanori Kawasaki, Nobuhiro Takasugi, Hajime Imai, Yoshiyuki Ishihara, Munenori Okubo, Shigekiyo Takahashi, Hironobu Sato, Kazuhiko Nishigaki, Genzou Takemura, Shinya Minatoguchi

**Affiliations:** 1 Department of Cardiology, Gifu University Graduate School of Medicine, Gifu, Japan

**Keywords:** Atrial fibrillation, Tissue characterization, Transesophageal echocardiography, Integrated backscatter

## Abstract

**Background:**

It is recognized that one of the causes of atrial fibrillation (AF) is pathological degeneration of the left atrium (LA). However, prospective study that elucidated the relationship between the incidence of persistent AF and pathological degeneration has not been performed. The purpose of this study was to elucidate the usefulness of integrated backscatter (IBS) values for the prediction of progression from paroxysmal AF (PAF) to persistent AF.

**Methods:**

We measured IBS values of the entire LA wall at 5 mm intervals (except the posterior wall) in 27 patients with paroxysmal AF and evaluated progression to persistent AF for three years. IBS values were acquired with transesophageal echocardiography (TEE) using a 4–7 MHz transducer. IBS values were calculated as the average power of the backscattered signal from regions of interest (ROI). Each IBS value was color-coded to construct three dimensional maps.

**Results:**

Average IBS values of total voxels in color-coded maps in the persistent AF group were significantly greater than those in the non-persistent AF group (25.8 ± 5.0 dB vs. 17.4 ± 10.2 dB, p = 0.047), whereas there was no significant difference in LA diameter between the persistent AF and the non-persistent AF group. There was significant difference in persistent AF-free survival after the baseline measurements in the subjects stratified by IBS value (<20 dB versus ≥20 dB) (univariate Cox regression analysis: hazard ratio: 8.74, p =0.046).

**Conclusion:**

Using IBS values measured by TEE, we can identify an increase in atrial degeneration that may predict the occurrence of persistent AF before LA dilation.

## Introduction

Atrial fibrillation (AF) is the most common sustained cardiac arrhythmia and is associated with increased mortality and morbidity [1,2]. Left atrial (LA) structural, functional and pathological remodeling occurs in response to conditions such as tachycardia, volume and/or pressure overload, diastolic dysfunction, ischemia and valvular disease [3]. Risk stratification for the development of AF based on LA remodeling may have a major public health impact. Parameters that reflect LA size such as LA dimension (LAD) and LA volume (LAV) are markers of elevated left ventricular filling pressure and have been proposed as predictors of AF [4-6]. However, there have been few studies that examined whether LA pathological degeneration predicts the risk of AF, because assessment of LA function has been limited by the lack of appropriate methods. An autopsy study reported increased amounts of fibrosis in the atria of patients with AF compared to those in sinus rhythm [7]. Another study reported that some of the histological features of the left atrium in patients with AF were an increase in interstitial tissue with infiltration of fatty tissue, interstitial fibrosis, and disruption of atrial muscle [8,9].

To predict the occurrence of AF, it is important to evaluate the degeneration of LA tissue. Investigation of LA tissue characteristics based on atrial biopsies demonstrated abnormal histological features in multiple biopsy specimens in all patients with lone AF [10]. However, atrial biopsy requires considerable skill and often results in complications. Therefore, there is no appropriate method to evaluate degeneration of the LA routinely in the clinical setting.

A promising technique to evaluate tissue characteristics *in vivo* is integrated backscatter (IBS) ultrasound. We have shown that IBS values of left atrium obtained from transesophageal echocardiography (TEE) reflected an increase in atrial degeneration that may predict the occurrence of AF [11]. We demonstrated that the optimal cutoffs of IBS values for the prediction of occurrence of AF were 20 dB [11].

The purpose of this study was to elucidate the usefulness of IBS values measured by TEE for the prediction of progression from paroxysmal AF (PAF) to persistent AF.

## Methods

### Subjects and study protocol

This was a prospective study that elucidated diagnostic accuracies of IBS values for the prediction of the progression from PAF to persistent AF. From January 2006 until February 2007, 27 patients with PAF who underwent TEE in order to evaluate valvular disease or the presence of thrombus in the cardiac chambers were enrolled in the present study. Exclusion criteria include unstable angina or myocardial infarction within the previous three months, an ejection fraction < 30 %, chronic heart failure (≥ NYHA III), mitral valve stenosis (mitral valve area < 2 cm^2^), severe mitral regurgitation or prevalent esophageal varices. Atrial natriuretic peptide (ANP) and brain natriuretic peptide (BNP) were measured and conventional transthoracic echocardiography (TTE) was performed at the same time as TEE. Risk factors for coronary artery disease were evaluated in each patient including diabetes mellitus (medication dependent, including oral hypoglycemic drugs and insulin), hypertension (medication-dependent only), smoking status (current smoker or quit <6 months before the study) and dyslipidemia (medication-dependent only). We performed monthly electrocardiogram (ECG) examinations in all enrolled patients, regardless of symptoms of arrhythmia during the follow-up period. All patients who had palpitations and/or symptoms of arrhythmia underwent both ECG and Holter ECG examinations to confirm progression from PAF to persistent AF. Persistent AF was defined as AF that was sustained >7 days or lasts <7 days but necessitates pharmacologic or electrical cardioversion [12,13].

The progression from PAF to persistent AF was confirmed by Holter ECG examination in all patients. We defined the time of progression from PAF to persistent AF as the month when the Holter ECG confirmed the progression from PAF to persistent AF. The present study was approved by the ethics committee of our institution and informed consent was obtained from all patients before enrollment.

### Echocardiography and IBS measurement in the clinical study

**Figure 1:**
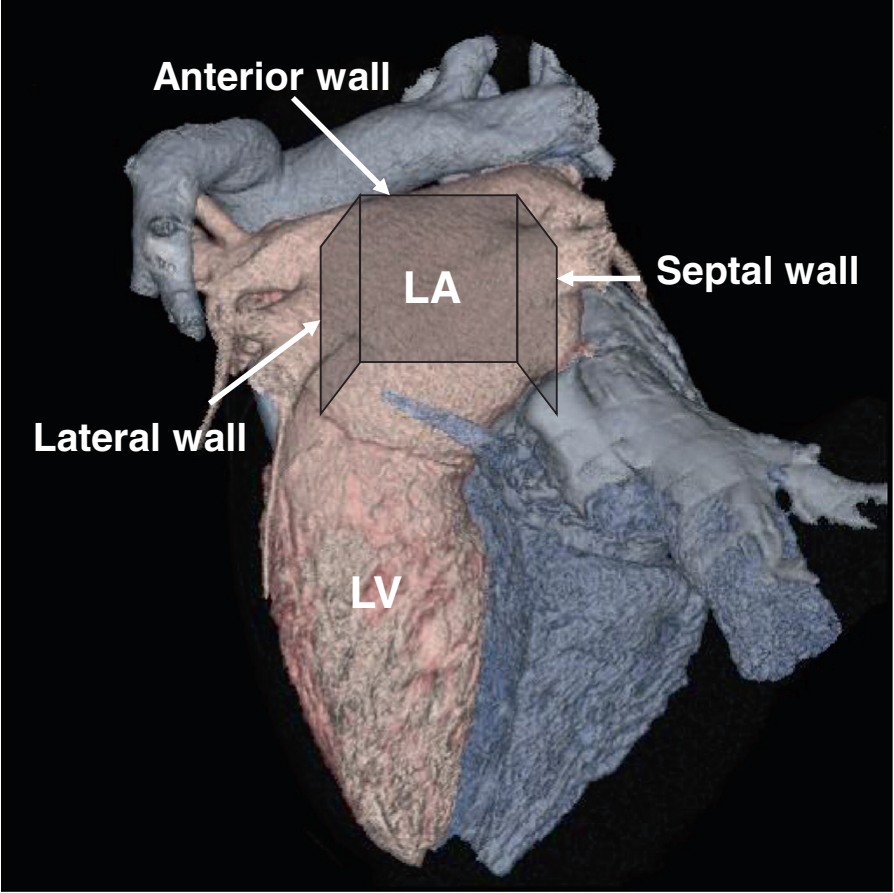
**Measurement site of left atrium.** Schematic image of the analyzed site (square). Lateral, anterior and septal walls of the left atrium were analyzed. LA: left atrium. LV: left ventricle.

Left ventricular end-diastolic dimension (LVEDD), left ventricular ejection fraction (LVEF) and LAD were measured by transthoracic echocardiography (SONOS 7500, Philips Medical Systems, Andover, MA). LA volume was calculated using an ellipsoid model that was reported in recommendation for chamber quantification [14]. After those measurements, TEE was performed with a 4–7 MHz multiplane transducer with a 7.4 mm diameter pediatric probe to diminish patient discomfort with the console of TTE (SONOS 7500, Philips Medical Systems, Andover, MA). The oropharynx was anesthetized with lidocaine before TEE. After the cardiac examination, images of the left atrium were depicted and IBS values of entire lateral, septal and anterior walls of the left atrium were measured at 5 mm intervals with a small region of interest (ROI) (6 x 6 pixels, 0.3 x 0.3 mm) set at each location as described previously (Figure 1) [11]. We set the time gain compensation at 70 dB and the lateral gain compensation at 70 dB at every measurement. At this setting, the IBS value of a stainless steel needle at a distance of 4 cm from the transducer was 63 dB. When the frequency of the transducer was 4–7 MHz, the resolution was approximately 0.2-0.4 mm assuming a sound velocity in tissue of 1,540 m/sec. The posterior wall (an angle span of 180 degrees between −90˚ and +90˚) was excluded from the analysis because the wall was affected by the diffraction and reverberation phenomena due to the short distance from the probe and angle dependency [15]. Therefore, IBS evaluation was performed using just the lateral, septal and anterior walls (Figure 1). The IBS values of the LA wall were corrected (corrected IBS: cIBS) by subtracting the IBS values in the LA cavity near the LA wall. We also evaluated relationship between variance of cIBS values and the progression from PAF to persistent AF because it was possible that heterogeneity of the degeneration of LA tissue affected the progression.

### Construction of three-dimensional color-coded maps of left atrium

Each IBS value was color coded to construct three-dimensional (3D) IBS maps of the entire lateral, septal and anterior walls of the left atrium. Three-dimensional image construction of LA tissue degeneration was performed by computer software (T3D, Fortner Research LLC, Sterling, Virginia). We employed mean cIBS values of the entire LA wall (except the posterior wall) to evaluate LA degeneration.

### Reproducibility and reliability of data

We previously determined inter-observer variability of cIBS values in 30 TEE recordings that were measured by two observers at randomly selected cross-sections. The inter-observer variability of cIBS values was 1.1 ± 3.0 % [16]. The inter-observer correlation coefficient was 0.98 for cIBS values. Likewise, we determined intra-observer variability of cIBS values in 30 TEE recordings that were measured two times by one observer at randomly selected cross-sections. The intra-observer variability of cIBS values was 0.5 ± 3.2 %. The intra-observer correlation coefficient was 0.98 for IBS values [17]. We determined inter-observer variability of average cIBS values in 20 randomly selected color-coded maps that were measured by two observers. The intra-observer variability of the average cIBS values in color-coded maps was 2.4 ± 2.3 %. The inter-observer agreements of average cIBS values in color-coded maps determined by linear regression was excellent (r =0.94, p < 0.001).

### Statistical Analyses

Numerical data are expressed as the mean ± one standard deviation. The Kolmogorov-Smirnov test was used to determine if data were normally distributed. If data were not normally distributed testing for significant differences of each parameter was performed with a Mann–Whitney U test between two groups. Categorical data were summarized as percentages and compared using a Chi-square test. Survival curves were plotted by the Kaplan-Meier method and hazard ratios were calculated by univariate Cox regression analysis. A p value <0.05 was considered to be significant. Statistical analyses were performed using Stat View version 5.0 (SAS Institute Inc, Cray, NC, USA).

## Results

### Patient characteristics

The patients’ clinical characteristics at baseline are listed in Table 1. During a follow-up period of three years (median: 37 months, 25th percentile: 34 months, 75th percentile: 40 months), 7 of 28 (25.0 %) subjects developed Holter electrocardiographically-confirmed persistent AF. There were no significant differences between the two groups in the age, history of diabetes mellitus, history of dyslipidemia, history of hypertension, current smoking and concomitant medication use. There were tendencies that ANP, BNP and D-dimer in the persistent AF group were higher than those in the non-persistent AF group. However, there were no significant differences between the groups.

**Table 1:** Demographics and baseline characteristics of the patients

	**progression (−)**	**progression (+)**	**p value**
	**(n = 21)**	**(n = 7)**	
Men, n (%)	18 (86)	6 (86)	>0.99
Age, y	59 ± 14	63 ± 13	0.50
Laboratory parameters, (mg/dl)			
ANP	38 ± 42	65 ± 23	0.11
BNP	77 ± 172	139 ± 136	0.40
D-dimer	0.63 ± 0.40	1.65 ± 2.05	0.56
C-reactive protein	1.06 ± 2.07	1.98 ± 4.94	0.93
Clinical history, n (%)			
Hypertension	11 (52)	4 (57)	>0.99
Diabetes mellitus type 2	7 (33)	3 (43)	0.67
Current smoker	3 (14)	1 (14)	>0.99
Dyslipidemia	8 (38)	1 (14)	0.37
Medication, n (%)			
Statins	3 (14)	0 (0)	0.55
Antiarrhythmic medication	3 (14)	1 (14)	>0.99
Calcium channel blockers	6 (29)	1 (14)	0.64
ß-blockers	6 (29)	1 (14)	0.64
ACE inhibitors or ARBs	8 (38)	3 (43)	>0.99

Plus-minus values are mean ± one standard deviation. CAF: chronic atrial fibrillation. ANP: atrial natriuretic peptide. BNP: brain natriuretic peptide. ACE: Angiotensin converting enzyme. ARB: Angiotensin II receptor blocker.

### Ultrasound parameters

**Figure 2:**
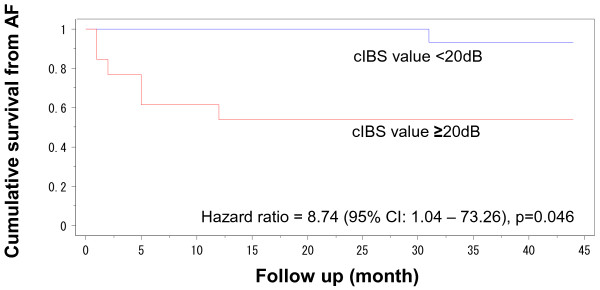
**Cumulative survival free from persistent atrial fibrillation.** Kaplan-Meier curves show the difference in persistent atrial fibrillation-free survival after the baseline measurements in the subjects stratified by cIBS: corrected integrated backscatter (cIBS) value (<20 dB versus ≥20 dB)CI: confidence interval.

Ultrasound parameters are shown in Table 2. The cIBS values in the AF group (25.8 ± 5.0 mm) was significantly greater than those in the non-AF group (17.4 ± 10.2 mm, p =0.047). There were no significant differences of LAD, LA volume, LVEDD, LVEF and LAA peak velocity between the two groups. Kaplan-Meier curves in Figure 2 show the difference in persistent AF-free survival after the baseline measurements in the subjects stratified by cIBS value (<20 dB versus ≥20 dB) (univariate Cox regression analysis: hazard ratio: 8.74, 95 % CI: 1.04 - 73.26, p =0.046). Diagnostic accuracies of corrected IBS value for persistent AF were shown in Table 3. There was no relationship between the variance of cIBS values between the progression-positive group and negative group (29.8 ± 13.8 vs. 28.7 ± 21.2, respectively, p =0.89).

**Table 2:** Ultrasound parameters of the patients

	**progression (−)**	**progression (+)**	**p value**
	**(n = 21)**	**(n = 7)**	
LAD (mm)	37.8 ± 6.3	40.2 ± 6.3	0.40
LA volume (ml)	29.1 ± 12.1	39.5 ± 29.4	0.20
LVEDD (mm)	49.6 ± 7.5	48.7 ± 5.9	0.79
LVEF (%)	64.0 ± 8.5	64.9 ± 11.9	0.84
LAA peak velocity (cm/sec)	55.8 ± 21.3	48.1 ± 21.1	0.41
Corrected IBS value (dB)	17.4 ± 10.2	25.8 ± 5.0	0.047

Plus-minus values are mean ± one standard deviation. CAF: chronic atrial fibrillation. LAD: left atrial dimension. LVEDD: left ventricular end-diastolic dimension. LVEF: left ventricular ejection fraction. LAA: left atrial appendage.

**Table 3:** The diagnostic accuracies for predicting progression from paroxysmal atrial fibrillation to persistent atrial fibrillation

	**Sensitivity**	**Specificity**	**PPV**	**NPV**
IBS value (≥20 dB)	86 (73–99)	67 (50–84)	46 (28–64)	94 (85–100)

Data are percentages. Number in parentheses are 95 % confidence intervals. PPV: positive predictive value. NPV: Negative predictive value.

### Construction of three-dimensional color-coded maps of left atrium

**Figure 3:**
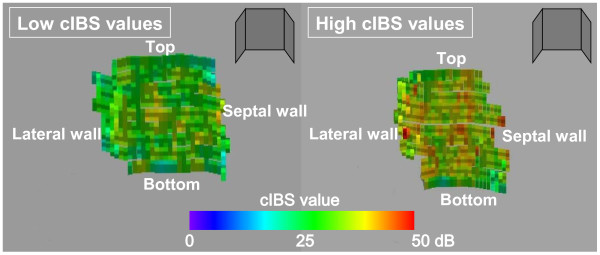
**Three-dimensional integrated backscatter color-coded maps of the left atrium.** The area of a high degree of degeneration (high corrected integrated backscatter values) was indicated by red and yellow colors and no or a low degree of degeneration (low corrected integrated backscatter values) was indicated by green and blue colors. cIBS: corrected integrated backscatter.

Three dimensional (3D) IBS-TEE color-coded images were consisted of a total of 228 ± 122 pixels in each atrium. The area with a high degree of degeneration was indicated by red and yellow colors and no or a low degree of degeneration was indicated by green and blue colors. The 3D IB-TEE images allowed visualization of LA degeneration as signified by a red color in the color-coded maps (Figure 3). By looking at these images, we were easily able to identify the location of areas of degeneration in the LA wall.

## Discussion

The findings in the present study demonstrated that cIBS values in patients that developed persistent AF were greater than those in patients that did not develop persistent AF. However, the LAD was similar in patients with and without persistent AF. These findings indicated that the LA myocardium in patients with persistent AF had already degenerated before enlargement of the left atrium. Therefore, we may predict the patients who were likely to progress from PAF to persistent AF by evaluating cIBS values of the left atrium. To the best of our knowledge, this is the first prospective study to elucidate clinically the relationship between tissue characteristics of the left atrium and the progression to persistent AF.

### Degeneration of LA tissue detected by integrated backscatter ultrasound

Ultrasound backscatter power is proportional to the difference of acoustic characteristic impedance that was determined by the density of tissue multiplied by the speed of sound. Because of the complex geometry of the left atrium, the acoustic characteristic impedance is highly variable. Based on these principles, we previously reported that it was possible to evaluate the degeneration of LA tissue by applying IBS analysis to TEE [11]. In a pathological comparison, the relative interstitial area increased as the cIBS values of the LA wall increased [11]. Cardiac remodeling, especially in the left atrium, is more pronounced in patients with AF. This explanation is supported by an autopsy study that demonstrated AF patients had increased fibrosis in the atria compared with patients in sinus rhythm [8]. Another study demonstrated that some of the histological features of the left atrium in AF patients were an increase in interstitial tissue with infiltration of fatty tissue, interstitial fibrosis, and disruption of atrial muscle [9]. The left atrium is more susceptible to fibrosis than the ventricle, although the precise mechanisms involved in the development of LA fibrosis are currently unknown [18]. The left atrium in the patients with persistent AF may be degenerated before the enlargement of the left atrium.

### Histological remodeling and functional remodeling

**Figure 4:**
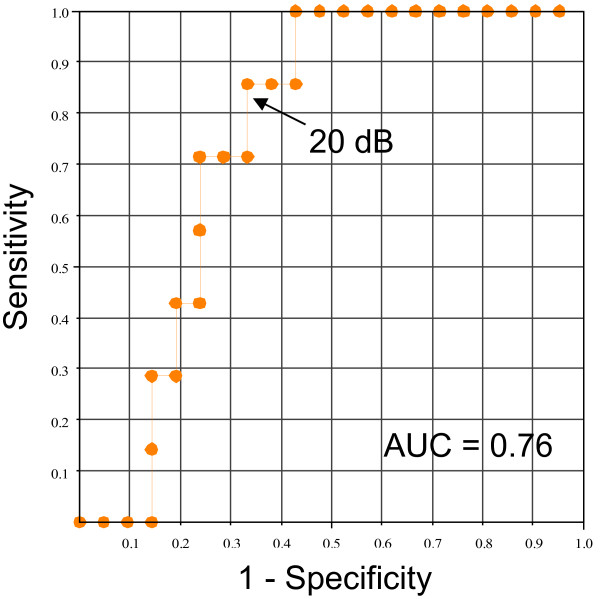
**Receiver operating characteristic curves analysis for predicting persistent atrial fibrillation.** AUC: area under the curve.

We previously demonstrated that there was histological remodeling of the left atrium in PAF patients before LA enlargement [11]. The stretched atrial tissue is associated with an increased arrhythmogenic activity and LA dilation, and this contributes to the perpetuation of AF [19-21]. Our other prospective study also demonstrated that baseline active LA emptying function (booster pump function) assessed in sinus rhythm was reduced regardless of LA size in patients that developed new-onset AF, and LA functional remodeling occurred prior to the first episode of AF [22]. Taken together, these findings indicate that both histological and functional LA remodeling occur prior to LA enlargement. Although, the relationship between the incidence of AF and histological remodeling has not been evaluated prospectively, we prospectively defined the relationship between the incidence of AF and functional remodeling. The present study showed that cIBS values of the left atrium could be useful to predict the patients who were likely to progress from PAF to persistent AF. We previously compared patients with and without AF and showed that the optimal cIBS cutoff value for the prediction of AF was 20 dB [11]. In that training study, the sensitivity and specificity for the prediction of AF were 79 % and 78 %, respectively [11]. In the present validation study, similar values of sensitivity and specificity for the prediction of AF were obtained (86 % and 67 %, respectively) (Figure 4).

### Clinical implications

Previous studies have reported that treatment with angiotensin converting enzyme inhibitors or angiotensin II receptor blockers can delay the progression of PAF to chronic AF [23-25]. The findings of the present study suggest that it may be possible to delay the progression of PAF to persistent AF by detecting the patients with high degree of LA degeneration and initiating treatment with angiotensin converting enzyme inhibitors or angiotensin II receptor blockers.

### Study limitations

There are several limitations of the present study. First, because the number of patients in our analysis was small and the statistical power was low, the significance of differences in echocardiographic parameters between the groups except for cIBS values could not be evaluated. A large-scale prospective follow-up study will be required in the future. Second, evaluation of echocardiographic parameters was performed only at baseline, although patients were followed-up for three years to determine the incidence of persistent AF. It is possible that the patient characteristics changed during the follow-up period of three years. Third, only patients with valvular or other cardiac disease were enrolled. Therefore, the findings of the present study may not be applicable to the general population. Finally, the number of incident AF cases may have been underestimated because some patients with persistent AF had no symptoms and only AF episodes that were confirmed by Holter ECG were considered an endpoint.

## Conclusions

Using cIBS values measured by IBS-TEE, we identified increased degeneration in the LA wall in patients with persistent AF, and this may be useful to predict the progression from PAF to persistent AF.

## Abbreviations

AF, Atrial Fibrillation; LA, Left Atrial; LAD, Left Atrial Dimension; LAV, Left Atrial Volume; IBS, Integrated Backscatter; TEE, Transesophageal Echocardiography; PAF, Paroxysmal Atrial Fibrillation; ANP, Atrial Natriuretic Peptide; BNP, Brain natriuretic Peptide; TTE, Transthoracic Echocardiography; ECG, Electrocardiogram; LVEDD, Left Ventricular End-Diastolic Dimension; LVEF, Left Ventricular Ejection Fraction; 3D, Three-Dimensional; cIBS, Corrected IBS.

## Authors’ contributions

TK and NT carried out subject recruitment and analyzed data. MK analyzed data and wrote the manuscript. HI, YI, MO, ST and HS performed integrated backscatter ultrasound analysis. KN, GT and SM analyzed data. All authors read and approved the final manuscript.

## Competing interests

The authors declare that they have no competing interests.
